# Using String Metrics to Improve the Design of Virtual Conversational Characters: Behavior Simulator Development Study

**DOI:** 10.2196/15349

**Published:** 2020-02-27

**Authors:** Santiago García-Carbajal, María Pipa-Muniz, Jose Luis Múgica

**Affiliations:** 1 Computer Science Department Universidad de Oviedo Gijón Spain; 2 Cabueñes Hospital Gijón Spain; 3 Signal Software SL Parque Científico Tecnológico de Gijón Gijón, Asturias Spain

**Keywords:** spoken interaction, string metrics, virtual conversational characters, serious games, e-learning

## Abstract

**Background:**

An emergency waiting room is a place where conflicts often arise. Nervous relatives in a hostile, unknown environment force security and medical staff to be ready to deal with some awkward situations. Additionally, it has been said that the medical interview is the first diagnostic and therapeutic tool, involving both intellectual and emotional skills on the part of the doctor. At the same time, it seems that there is something mysterious about interviewing that cannot be formalized or taught. In this context, virtual conversational characters (VCCs) are progressively present in most e-learning environments.

**Objective:**

In this study, we propose and develop a modular architecture for a VCC-based behavior simulator to be used as a tool for conflict avoidance training. Our behavior simulators are now being used in hospital environments, where training exercises must be easily designed and tested.

**Methods:**

We define training exercises as labeled, directed graphs that help an instructor in the design of complex training situations. In order to increase the perception of talking to a real person, the simulator must deal with a huge number of sentences that a VCC must understand and react to. These sentences are grouped into sets identified with a common label. Labels are then used to trigger changes in the active node of the graph that encodes the current state of the training exercise. As a consequence, we need to be able to map every sentence said by the human user into the set it belongs to, in a fast and robust way. In this work, we discuss two different existing string metrics, and compare them to one that we use to assess a designed exercise.

**Results:**

Based on the similarities found between different sets, the proposed metric provided valuable information about ill-defined exercises. We also described the environment in which our programs are being used and illustrated it with an example.

**Conclusions:**

Initially designed as a tool for training emergency room staff, our software could be of use in many other areas within the same environment. We are currently exploring the possibility of using it in speech therapy situations.

## Introduction

### Virtual Conversational Characters

The field of virtual conversational characters (VCCs) is an emerging research field that is growing in importance, both in industrial and academic applications. Our company started to include VCCs as a component of our simulators in 2014, mainly oriented to military and police environments, and recently it was proposed to migrate this type of simulator to hospital environments.

VCCs, also known as embodied conversational agents by Poggi et al [1], are 2D and 3D models of real persons that must be capable of human-like behavior. Apart from high-quality graphics, the most important characteristics that define VCCs are as follows:

1. Degree of embodiment: a full embodiment implies rendering a complete body. A talking head is an example of partial embodiment.
2. Believable talking: the VCC must be able to maintain a conversation with the human user. The most difficult problem to solve is to manage communication in a way that the human user does not perceive his or her dialogue partner as an emotionally numb agent.
3. Gesturing: nonverbal behavior is key when trying to solve the traditional lack of naturalness of VCCs. Nonverbal behavior can be introduced in one or both of the following ways:
  - Facial gesture: different models and taxonomies for facial movement have been proposed by Ekman and Friesen [2] and Martinez and Shichuan [3]. An excellent state of the artwork on facial expressions for VCCs is that of Ochs et al [4].
  - Body gesture: this usually involves hand and arm movements while talking, as included by Hartholt et al [5] in the virtual human toolkit.
4. Emotional behavior of the character: for a VCC, it is desirable not only to be able to maintain a conversation, but also to do so while showing some kind of personality, mood, or attitude.

In this work, we focus on the dialogue management problem. Involving a VCC in a meaningful conversation often implies huge knowledge databases, syntactic and semantic analysis, and the use of artificial intelligence techniques to achieve a convincing result. Designing conversational situations as graphs in the way described in García et al [6], we restrict the possible states of the dialogue, the sentences to be said by the VCC, and the sets of sentences that it will understand. This method does not decrease the applicability of our behavior simulator, as it is intended to be used in strictly constrained situations. Unfortunately, two problems arise when using such an approach: (1) the need for a huge number of similar but slightly different sentences to be said by the VCC if we want the agent not to appear too repetitive and (2) on the other hand, we want the VCC to be able to understand an order, question, or command expressed in as many ways as possible.

The first problem can be solved merely by including a high number of different ways to express what the VCC is going to say and by randomly picking one of them at execution time. The second problem requires the mapping of the expressions said by the human user to any of those the VCC can accept, converting it into the associated label, and delivering it to the situation manager, all within the execution time. This is where string metrics come into play, as a way of measuring the similarities between sentences said by the human user and the sets of expressions the VCC is expecting.

### Related Work

Related works include Rosmalen et al [7], where an existing serious game is extended to include a chatbot, as well as those related to the formalization and use of behavior trees by Johansson and Dell’Acqua [8], Isla [9], or Imbert and de Antonio [10], where COGNITIVA is proposed as an emotional architecture for VCCs. The most closely related works to that reported here are those of Hartholt et al [5] and Morie et al [11], where the virtual human toolkit is described. More recently, a framework for the rapid development of Spanish-speaking characters has been presented in Herrera et al [12].

In this context, our system's characteristics are as follows:

1. Full embodiment: our VCCs are complete human models rendered inside a realistic 3D scene.
2. We solve the dialogue management problem by defining our training situations as graphs and by introducing a statistical process of strings returned by the speech recognition library as a way of directing the evolution of the exercise.
3. Inclusion of a facial action code system, as described by Ekman and Friesen [2], as a way of manipulating the VCC's facial gesture.
4. Emotional behavior is based on an emotional engine that permits the design and testing of the underlying personality of the VCC, described in García et al [6], and is mainly oriented to the simulation of violent behaviors, as this has been the main application field of our software.

The rest of the paper is structured as follows:

1. The Environment section describes the context where string metrics are being used.
2. In the Situation Graphs section, we describe the component of the behavior simulator to be analyzed using string metrics.
3. The String Metrics section is devoted to the explanation of some string metrics and their comparison to the one we are using.
4. In the Graph Validation section, three different string metrics are applied to an example graph using our graph validation tool.
5. Finally, in the Conclusions and Future Work sections, we present the main achievements of our work and some possible future lines of research.

## Methods

### Environment

In this section, we describe the context in which we are using string metrics. Our simulators are designed to be used in conflict avoidance training contexts, including situations where a member of the security staff must ask a suspect for his or her identity card, begin the initial evaluation process of a patient, or deal with an annoyed relative. Such situations are characterized by the fact that there is a clear policy the trainee must follow in order to fulfill the exercise. Conversely, the VCC will have a small set of expected behaviors. Therefore, we need to build a believable VCC that is able to communicate in a clearly constrained scene.

Our tool lets the user give a formal description of the training exercise. The output of the tool is a directed graph in Graphviz format, following the description of Emden and North [13], that represents the current and possible states of the exercise and defines transitions from one state to another, in terms of the labels associated with each arc. The main components of our behavior simulator are as follows:

1. A situation graph, defining the exercise.
2. A set of sentences associated with each node of the graph. Whenever the situation enters a state, the system will randomly pick one of the sentences associated with that node. The higher the number of sentences, the lower the probability of repeating a sentence, while increasing the perception of talking to a real human.
3. One or various sets of sentences that the VCC must recognize when the graph is in a valid state.

Each node in the graph will be connected to one or more other nodes. The arcs representing these connections will be labeled with names like *Ask_For_ID*, which require an action from the human user. Each label will be associated with a set of sentences that the human user can say in order to trigger that transition. We keep this kind of information stored in files sharing the *.lang* extension. The other elements of the system are as follows:

1. An emotional engine that drives the emotional state and behavior of the VCC, as described in García et al [6].
2. A body language interpreter that is developed using the Microsoft Kinect sensor, which analyzes the body gesture of the human interacting with the simulator, in order to give advice about good or bad practices while interacting with real humans.

### Situation Graphs

Any one of our situation graphs will contain, at least, the following states:

1. Init: in this state, the system performs some basic tasks, such as graph file parsing, audio and graphical setup, and some initial calculations that increase performance, which will be explained in the Histogram Matching section.
2. Success: this state will be reached when the human performing the training exercise completes it in a satisfactory manner.
3. Failure: the opposite of the Success state.

In order to clearly state the role of the situation graph, we will describe an unreal, simple training situation with its associated states and sets of sentences. Describing a medical interview in terms of one of our situation graphs generates a huge image, too big for the illustrative purposes of this section.

**Figure 1:**
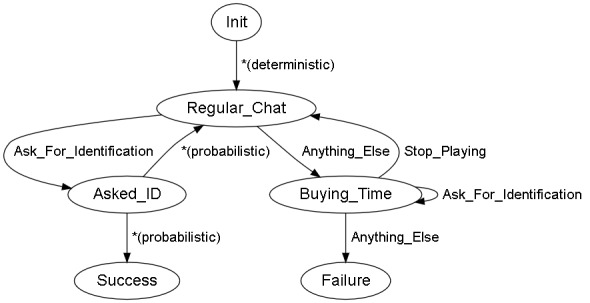
Simple situation graph. Init: the state in which the system performs some basic tasks. Success: this state will be reached when the human performing the training exercise completes it in a satisfactory manner. Failure: the opposite of the Success state. *Regular_Chat*: as soon as the exercise starts, the scene enters this state, with the virtual conversational character (VCC) engaging in small talk. *Asked_ID*: the situation enters this state if the user says one of the sentences associated with the *Ask_For_Identification* label; when in this state, the VCC will probabilistically decide to collaborate or not, showing ID, or returning to *Regular_Chat*. The former means reaching the Success state. The latter means that the VCC refused to obey and show their ID card. In practice, this implies remaining in the same state, *Regular_Chat*. *Buying_Time*: if the user does not ask for identification, the scene enters a dumb state, with the VCC trying to escape. If the user continues asking for ID, the situation reaches an impasse. To return to *Regular_Chat*, the security guard must warn the VCC about trying to escape. Any other kind of conversation triggers a Failure.

We have a situation where the behavior simulator, once initialized, will present the user with a VCC. The goal is to obtain their identification card, to avoid the start of a fight, or to prevent the individual from running away from the scene. The situation graph is shown in Figure 1.

Associated with each state in the graph are a stored set of sentences that the VCC will keep saying until the answer received from the user triggers a transition to another state. The system stores these sets in files named after the state they are associated with; all of them share the *.talk* extension in their file names. Based on the contents of the *.talk* files, the behavior simulator will keep the VCC saying a sentence picked at random from those associated with the current active node of the situation graph. The behavior simulator will also try to map what the human user says into the labels that can trigger a transition from the current state to any other.

At the moment of writing this paper, we have designed four different training situations, or exercises, each with their own learning goals:

1. A lost child in the waiting room: the goals are to discover where the child came from and what she is doing at the hospital.
2. An aggressive young man under the influence of drugs: the goal is to gain time while security personnel arrive.
3. An elderly woman with cognitive impairment: the goals are to make an initial assessment of the woman's condition and to reassure her.
4. A nervous young lady asking for information about one of her relatives: the goal is to convince her to leave the area and retire to the waiting room.

**Figure 2:**
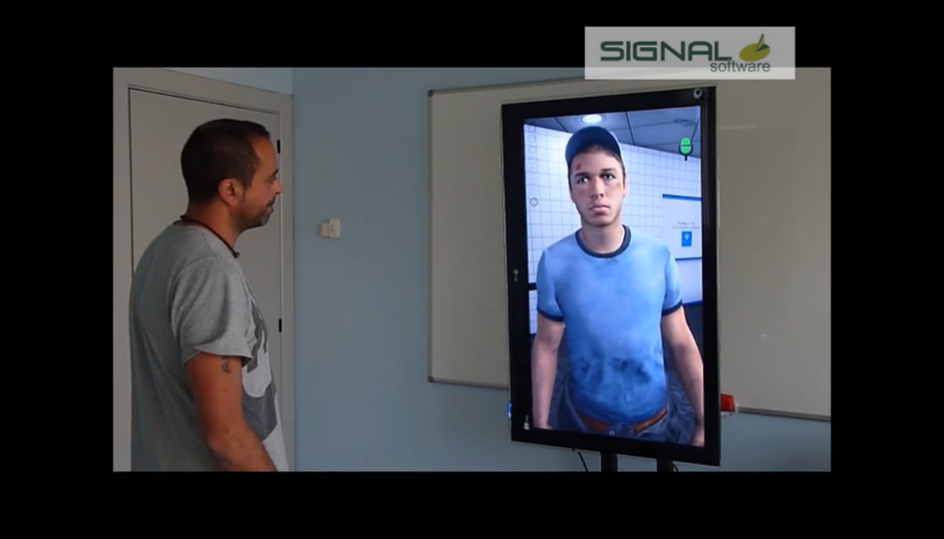
A user facing the system a moment after starting a training exercise.

Figure 2 shows the system a moment after starting one of these training exercises. The system can be run in silent mode, showing sentences said by the VCC only on the screen in text, and the microphone can be disconnected to allow input of sentences using the keyboard.

### String Metrics

#### Overview

In order to trigger transitions from one state of the graph to another, we need some kind of metric to evaluate the distance between the string returned by the speech recognition library and all the strings that are acceptable for the current state. There is a large number of metrics that can be used to measure the difference between pairs of strings. In this section, we compare two different well-known metrics—that proposed by Levenshtein [14] and the Gestalt pattern-matching algorithm proposed by Ratcliff and Metzener [15]—when used for our purposes and justify the use of our own metric, which we will call histogram matching.

String distance functions or string similarity metrics are defined between two strings, for example, s and t. Distance functions map a pair of strings s and t to a real number r, where a smaller value of r indicates greater similarity between s and t. Similarity functions are analogous to distance functions, except that larger values indicate greater similarity.

#### Levenshtein Distance

One important class of distance functions are edit distances, in which distance is the cost of the best sequence of edit operations that converts s to t. Typical edit operations are character insertion, deletion, and substitution, and each operation much be assigned a cost. Levenshtein distance is defined in Levenshtein [14]. However, even in its normalized version proposed by Yujian and Bo [16], it is not useful for us, as it gives high values to pairs of strings that are a word-by-word permutation of the original, for example, “Don't resist, please” and “Please, don't resist.” See Table 1 for results.

**Table 1:** Levenshtein distances for the strings s1, s2, and s3.

String	String, Levenshtein distance		
	s1^a^	s2^b^	s3^c^
s1	0	14	14
s2	14	0	16
s3	14	16	0

^a^s1: “Please show me your ID.”

^b^s2: “Show me your ID please.”

^c^s3: “Your ID. Show it to me.”

#### Gestalt Pattern Matching

Ratcliff and Metzener’s pattern-matching algorithm [15] has been described as a wild-card search process without wild cards. The algorithm builds its own wild cards, based on the matches found between two strings, s and t. First, the algorithm examines s and t and locates the largest common subsequence between them. It then uses this group of characters as an anchor between s and t. Any group of characters found to the left or the right of this anchor is placed on a stack for further examination. The procedure is repeated for all substrings on the stack until it is empty.

The returned value is twice the number of characters found in common, divided by the total number of characters in the two strings; the score is returned as an integer, reflecting a percentage match. We are currently using the SequenceMatcher version of Ratcliff's algorithm, included in the difflib package from Python, version 3.7 (Python Software Foundation), that returns a real number instead.

#### Histogram Matching

**Figure 3:**
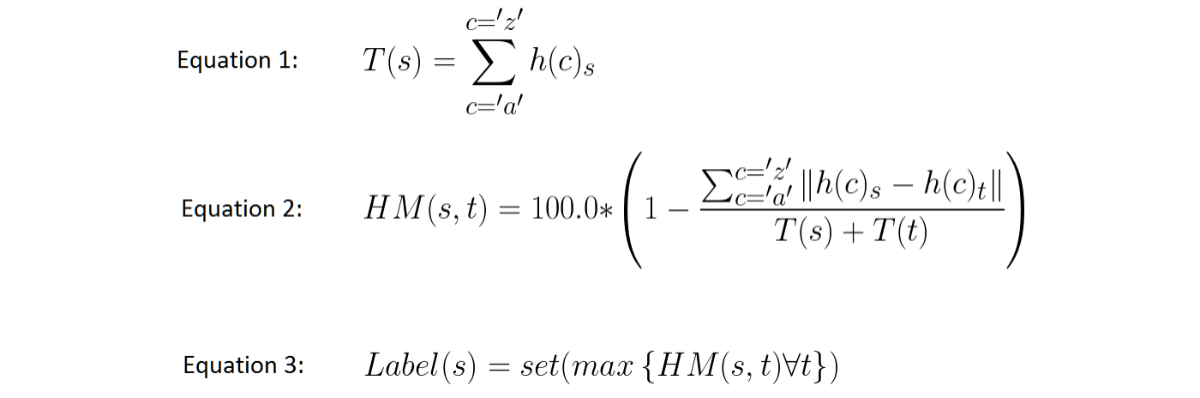
Equations for our histogram-matching metric.

We will now describe the numerical procedure that lets us assign a label to any string returned from speech recognition libraries, such as Microsoft Speech Application Programming Interface (API). When the exercise starts, we take each *.lang* file, and for each sentence we perform the following procedure (see Figure 3, Equation 1):

1. Convert the sentence to lowercase letters, discarding any punctuation marks.
2. Calculate the number of letter “a”s, “b”s, etc, that the sentence contains. This array is what we call a letter histogram. Letter histograms for every single possible sentence that the human user can potentially say are calculated and stored before the exercise starts.
3. Let h(c)s be the number of occurrences of character c inside string s.
4. Let T(s) be the sum of h(c)_s_ for each possible value of c inside string s.

When the exercise starts, we need to know the distance between the words said by the human user as well as all the sentences stored inside the *.lang* files. We define the histogram-matching function between strings s and t as expressed in Equation 2 (see Figure 3). In the histogram-matching formula (see Figure 3, Equation 2), s represents the sentence said by the human user, and t is each one of the sentences included in the *.lang* files that is associated with outgoing arcs from the current active node in the situation graph. The maximum of these values determines the label we assign to the sentence that was said and, eventually, a transition to another node inside the graph.

In Equation 3 (see Figure 3), set (t) is a function returning the set that t belongs to, and $set(t)$ is an outgoing arc.

## Results

### Data Evaluation

In this section we describe the results obtained when applying the three string metrics described in the Methods section to a set of sentences, and the process that we follow to validate a graph.

### Levenshtein Distance

Table 1 shows the value of Levenshtein distance for three different strings—s1, s2, and s3—with s2 being a word-by-word permutation of s1, and being very similar to s3, at least semantically. The strings s1, s2, and s3 stand for “Please show me your ID,” “Show me your ID please,” and “Your ID. Show it to me,” respectively.

As we do not process commas, nor any other punctuation marks, s1 and s2 should be equivalent sentences for our system, and the distance between s3 and the others should be minimal. We are showing not-normalized values here, but it can be seen that the distance between s1 and s2 is not equal to zero, forcing us to include all the valid permutations of a sentence in the respective *.lang* file if we want the VCC to understand all of them. This renders the Levenshtein distance metric inappropriate for our labeling needs.

### Gestalt Pattern Matching

Table 2 shows the values of the Gestalt pattern-matching algorithm by Ratcliff and Metzener [15] when applied to s1, s2, and s3. It returns a 100% similarity value over the main diagonal, as expected, but the reported value is not symmetric for s2 and s3. Additionally, it gives a similarity value of 68% between s1 and s2, too low for a pair of sentences that must be considered equivalent for our system.

**Table 2:** Python SequenceMatcher similarities for the strings s1, s2, and s3, based on the Gestalt pattern-matching algorithm.

String	String, SequenceMatcher similarity		
	s1^a^	s2^b^	s3^c^
s1	100.0	68.18	32.55
s2	68.18	100.0	41.86
s3	32.55	46.51	100.0

^a^s1: “Please show me your ID.”

^b^s2: “Show me your ID please.”

^c^s3: “Your ID. Show it to me.”

### Histogram Matching

Table 3 shows histogram-matching values between the strings s1, s2, and s3. The main diagonal values are 100%, as expected, but we also see total similarity between s1 and s2. Reported similarity between s1 and s3, and between s2 and s3, is higher than 70%, which is far from being an almost complete match, but significantly higher than the value reported by the Gestalt pattern algorithm (32%).

**Figure 4:**
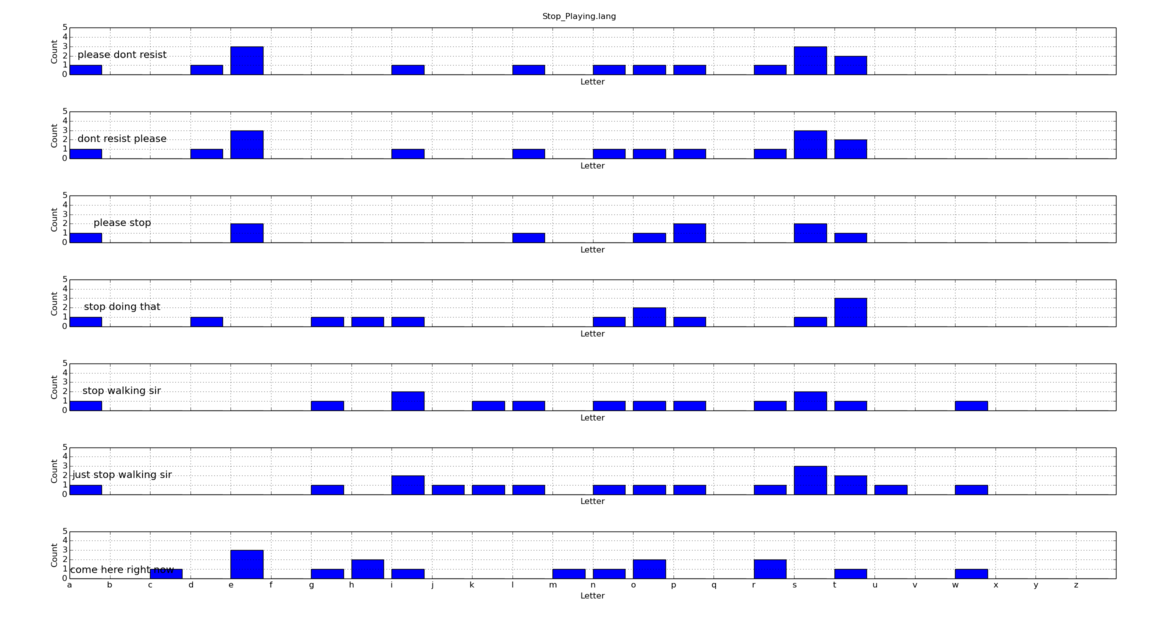
Letter histogram for the *Stop_Playing.lang* file.

**Figure 5:**
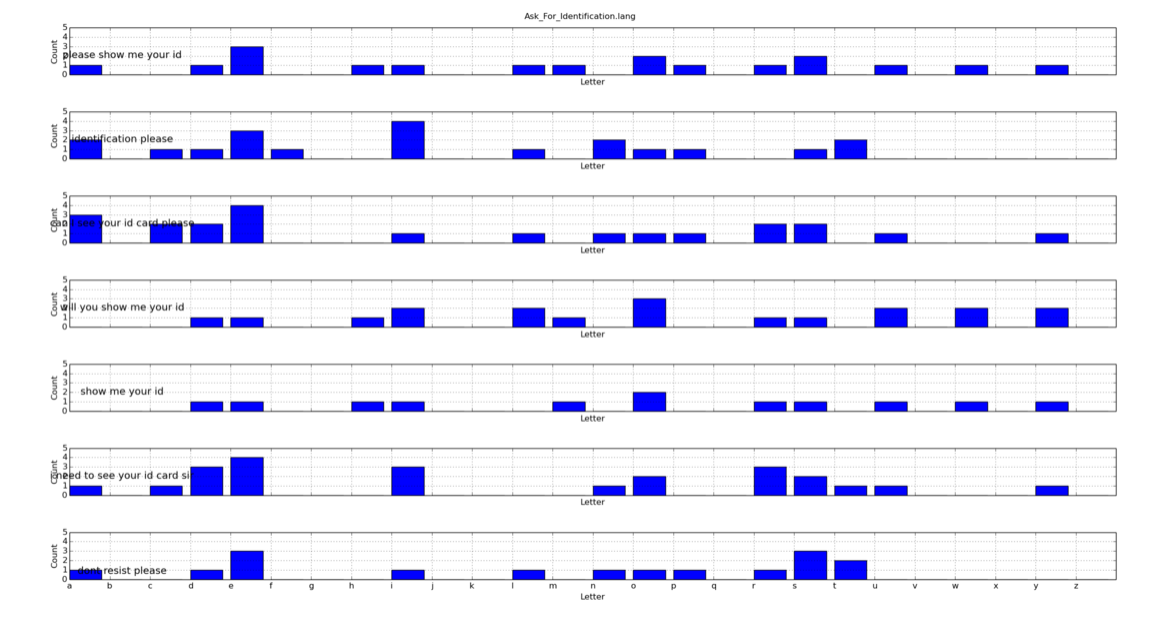
Letter histogram for the *Ask_For_Identification.lang* file.

Figures 4 and 5 show the letter histograms associated with every sentence included inside the *Stop_Playing.lang* and *Ask_For_Identification.lang* files.

The example has been intentionally kept simple but practical, in order to explain how histogram matching works. Similarities between the different sentences associated with the same label (ie, inside the same set) present no practical problem. Problems arise when, for two different labels associated with arcs that come out of the same graph node, any of the sentences included in the corresponding files can be misunderstood as any of those defined for a different transition. In other words, the distance between each of the sentences included in any *.lang* file and those included in a *.lang* file whose initial graph node is the same, should be as large as possible, in order to have the least confusing exercise definition. Additionally, as a reinforcement factor, we also count the number of blank spaces included in the sentence, to avoid strange coincidences that would confuse the system.

In the exercise we are using as an example, there is only one situation to analyze—there are three different arcs coming out from the *Buying_Time* state, and we need the triggering sentences for these arcs to be as different as possible: (1) *Stop_Playing*, (2) *Ask_For_Identification*, and (2) *Anything_Else*.

Of these three transitions (ie, labels), only the first two are interesting, as *Anything_Else* is a special case that we will assign if it is not possible to assign any of the others, up to a defined tolerance. We mark this kind of label, leaving the corresponding *.lang* file almost empty, containing only a # symbol. Therefore, it is clear that the exercise would be ill defined if any sentence inside *Stop_Playing.lang* is too similar to any of the sentences included in *Ask_For_Identification.lang*. Our exercise validation tool analyzes this kind of situation and highlights potentially conflicting labels, sentences, and states. The example is analyzed in the Graph Validation section

**Table 3:** Histogram-matching similarities for the strings s1, s2, and s3.

String	String, histogram-matching similarity		
	s1^a^	s2^b^	s3^c^
s1	100.0	100.0	70.5
s2	100.0	100.0	70.5
s3	70.5	70.5	100.0

^a^s1: “Please show me your ID.”

^b^s2: “Show me your ID please.”

^c^s3: “Your ID. Show it to me.”

### Graph Validation

For the purpose of explaining how graph validation works, we intentionally added the following sentence to the *Ask_For_Identification* set file: “Don't resist, please.”

**Figure 6:**
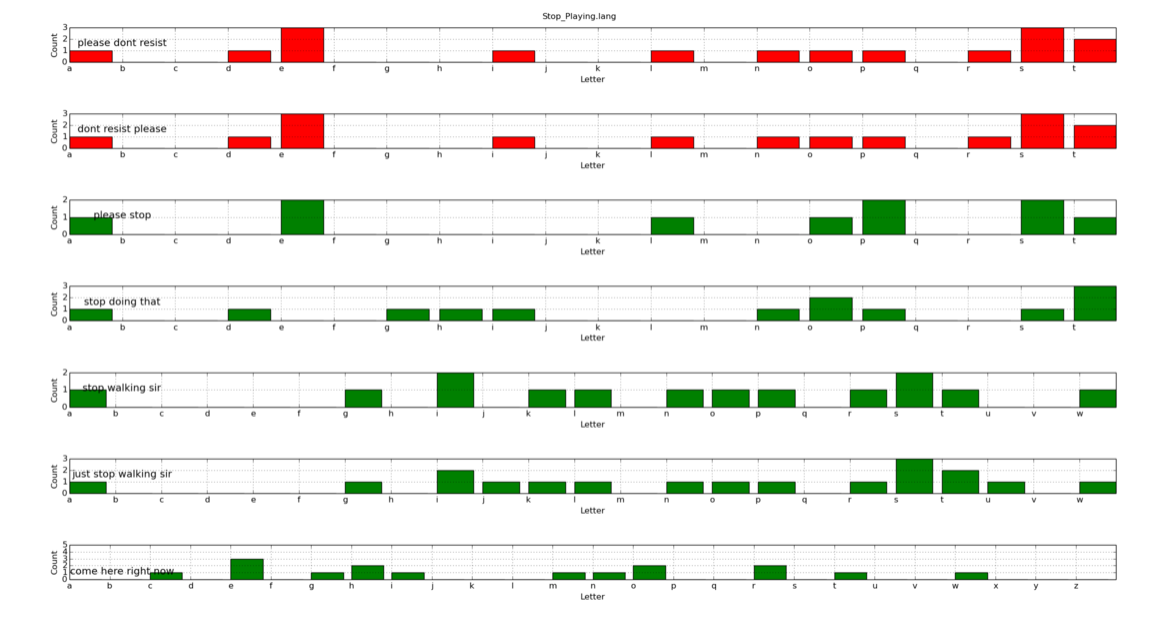
Processed histogram for the *Stop_Playing.lang* file.

**Figure 7:**
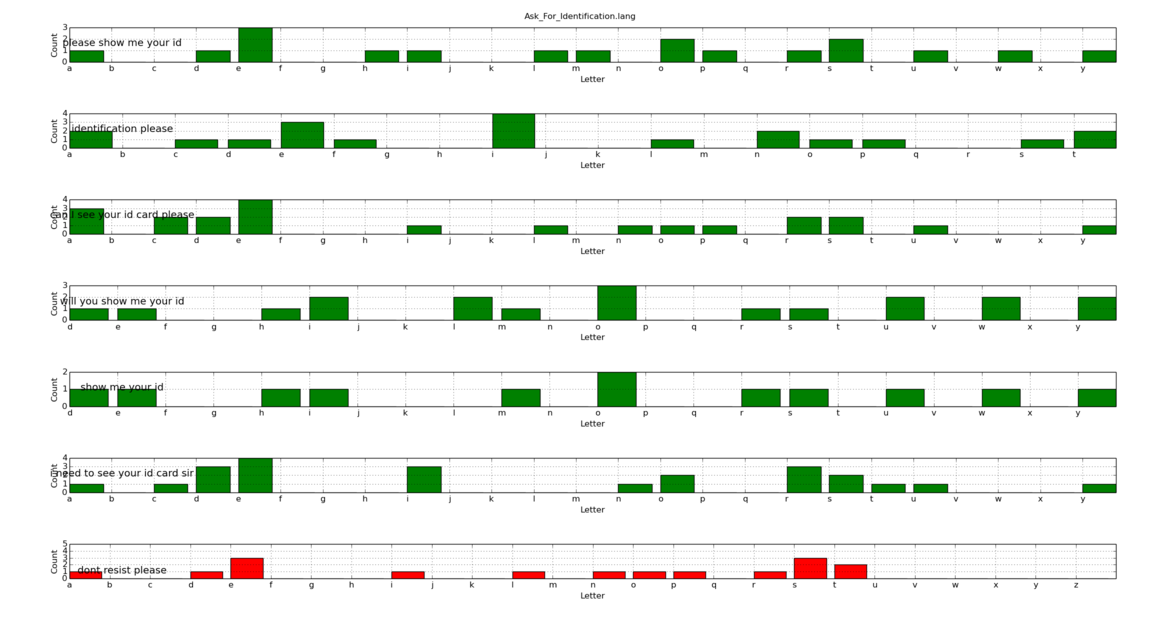
Processed histogram for the *Ask_For_Identification.lang* file.

Besides the lack of utility of such a sentence inside the file that stores different ways of asking a person for his or her hospital identity card, this sentence causes problems: when it is said by the human user, it would lead to ambiguity. In Figures 6 and 7, letter histograms for “Don't resist, please” and “Please, don't resist” are highlighted in red; this is not because of the absolute similarity between them, but because of their similarity to this extra sentence included in another *.lang* file, which would render the system unable to decide which transition is the correct one to be triggered.

The system informs us that sentences highlighted in red can confuse the situation manager when pronounced by the trainee. In this case, the solution is straightforward, as we have artificially generated the problem. The problem is solved simply by removing the extra sentence from the *Ask_For_Identification.lang* file. However, in more complex situations, the person in charge of the exercise design should look for alternatives.

**Figure 8:**
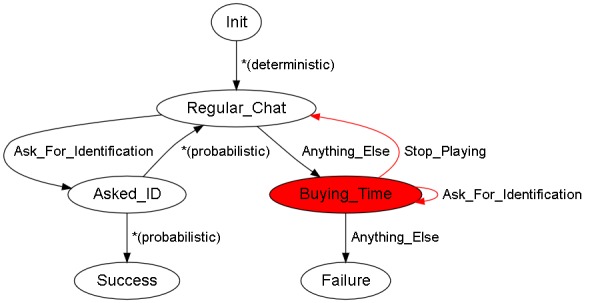
Validated graph. Init: the state in which the system performs some basic tasks. Success: this state will be reached when the human performing the training exercise completes it in a satisfactory manner. Failure: the opposite of the Success state. *Regular_Chat*: as soon as the exercise starts, the scene enters this state, with the virtual conversational character (VCC) engaging in small talk. Asked_ID: the situation enters this state if the user says one of the sentences associated with the *Ask_For_Identification* label; when in this state, the VCC will probabilistically decide to collaborate or not, showing ID, or returning to *Regular_Chat*. The former means reaching the Success state. The latter means that the VCC refused to obey and show their ID card. In practice, this implies remaining in the same state, *Regular_Chat*. *Buying_Time*: if the user does not ask for identification, the scene enters a dumb state, with the VCC trying to escape. If the user continues asking for ID, the situation reaches an impasse. To return to *Regular_Chat*, the security guard must warn the VCC about trying to escape. Any other kind of conversation triggers a Failure.

After determining all the conflicting labels, our graph validation tool also marks in red each graph node with ill-defined outgoing arcs, helping in the identification and fixing of such problems. The output is a file in the Graphviz format: see Figure 8, where the graph for the working exercise is colored to highlight problematic nodes. An arc whose arrow is highlighted in red means that triggering transitions from the source state is not possible. Nonproblematic arcs are highlighted in green. The goal is to rewrite the sentences associated with each arc or to modify the graph definition of the exercise until no ambiguity is detected by the tool.

The use of the string metric defined in this paper is not mandatory. In fact, the user can choose one of the following string metrics and select the one that guarantees a better exercise definition to be used by the behavior simulator: (1) Levenshtein distance, as defined by Levenshtein [14], (2) Gestalt pattern matching [15], (3) histogram matching, as proposed in this paper, (4) Damerau-Levenshtein distance [17], or (5) Jaro-Winkler distance [18].

**Figure 9:**
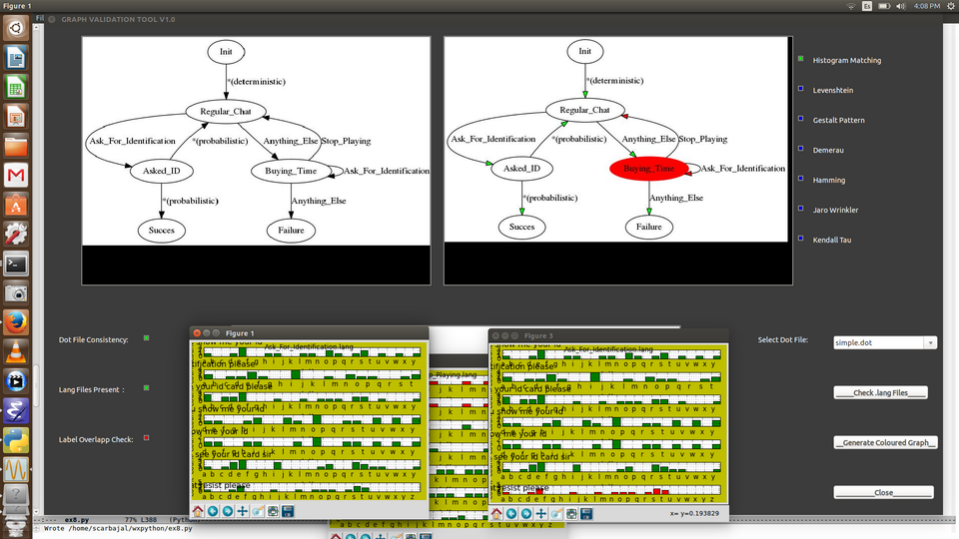
The main window of the graph validation tool. From this window, the user can (1) choose a Graphviz (.dot file) example to be analyzed, (2) obtain a graphic representation of the training exercise that it encodes, (3) select one of the available string metrics, (4) view letter histograms for each label, and (5) generate a .pdf file summarizing all the problems encountered during the analysis of the graph.

The explanation of each of these string metrics is outside the scope of this paper. In practice, we use the graph validation tool to choose a string metric that guarantees the absence of ambiguities when the simulator is running. If none of them can guarantee such a condition, the *.lang* files must be modified. The main window of the graph validation tool is pictured in Figure 9.

## Discussion

We have developed a system for the fast design and testing of conflict avoidance situations, involving interactions between humans and VCCs. VCC-enhanced simulators present many advantages for multimodal communication, but also have the disadvantage of dealing with complex processes in order to provide effective verbal communication between VCCs and the human user. Speech recognition software is available and working, but we needed a way to assign labels to the outputs produced by these APIs. As the number of possible sentences to be recognized is potentially huge, even for a simple training exercise, we decided to use string metrics as a way of labeling. We have developed a tool that, after designing a training exercise, analyzes the sets of sentences associated with each transition inside the situation graph, highlighting potential signs of ill-defined exercises. The tool is also used to check the existence of all the files needed for the system to work properly before the exercise starts and to dynamically change some settings, such as the minimal matching confidence level required for a positive match, once the simulation has begun. This is useful for cases when the speech recognition library is not working properly, due to suboptimal acoustic conditions of the environment or incorrect vocalization by the human user.

After trying several existing string metrics, we decided to design one of our own: histogram matching. Histogram matching does this work for us at a reasonable speed, as half of the needed calculations are performed as soon as the training exercise is defined and before the whole system is running. The method is working correctly for the exercises we have defined to date. As a result, we can anticipate and solve design problems in the training exercise definition process and improve collaborative work between instructors and our development team.

For the future, we are planning the development of a module that automatically assigns violence levels to the sentences included inside *.lang* files, as a function of the kind of vocabulary employed. There is another feature that has not yet been implemented, which would be very useful in the exercise definition process. That is, being able to check the exercise without the whole graphics system working, running only the speech recognition and language synthesis modules, and allowing the interactive visualization of the active node of the situation graph, histogram-matching level, emotional state of the VCC, etc.

Our software was initially designed to help in the training of staff working for emergency services within a hospital. We think that it could also prove useful in speech therapy as a way of visually representing the differences between any goal sentence and what a human user actually says. We have identified some works using serious games in this field, such as Grossinho et al [19] and Cagatay et al [20]. In this sense, no structural modifications should be needed on our software, just a different philosophy in the design of the training exercises. That is to say, we have a VCC that the human must interact with. The goal would be to speak as correctly as possible in order to, for example, make the VCC do some work for us.

We also think that VCCs, in general, and our system, in particular, can be useful in helping patients make informed decisions when asked about the treatment plan they prefer, as discussed in Sherwin et al [21].

## Abbreviations

API: application programming interface
VCC: virtual conversational character
